# Multisensory Integration in Non-Human Primates during a Sensory-Motor Task

**DOI:** 10.3389/fnhum.2013.00799

**Published:** 2013-11-20

**Authors:** Florian Lanz, Véronique Moret, Eric Michel Rouiller, Gérard Loquet

**Affiliations:** ^1^Domain of Physiology, Department of Medicine, Fribourg Cognition Center, University of Fribourg, Fribourg, Switzerland

**Keywords:** sensory-motor, detection task, non-human primate, facilitatory effect, electrophysiology

## Abstract

Daily our central nervous system receives inputs via several sensory modalities, processes them and integrates information in order to produce a suitable behavior. The amazing part is that such a multisensory integration brings all information into a unified percept. An approach to start investigating this property is to show that perception is better and faster when multimodal stimuli are used as compared to unimodal stimuli. This forms the first part of the present study conducted in a non-human primate’s model (*n* = 2) engaged in a detection sensory-motor task where visual and auditory stimuli were displayed individually or simultaneously. The measured parameters were the reaction time (RT) between stimulus and onset of arm movement, successes and errors percentages, as well as the evolution as a function of time of these parameters with training. As expected, RTs were shorter when the subjects were exposed to combined stimuli. The gains for both subjects were around 20 and 40 ms, as compared with the auditory and visual stimulus alone, respectively. Moreover the number of correct responses increased in response to bimodal stimuli. We interpreted such multisensory advantage through redundant signal effect which decreases perceptual ambiguity, increases speed of stimulus detection, and improves performance accuracy. The second part of the study presents single-unit recordings derived from the premotor cortex (PM) of the same subjects during the sensory-motor task. Response patterns to sensory/multisensory stimulation are documented and specific type proportions are reported. Characterization of bimodal neurons indicates a mechanism of audio-visual integration possibly through a decrease of inhibition. Nevertheless the neural processing leading to faster motor response from PM as a polysensory association cortical area remains still unclear.

## Introduction

Traditionally sensory modalities like vision, hearing, touch, pain, chemical senses, and others have been investigated mostly individually. However, the number of studies showing cross-modal integration (Calvert et al., 2000; Calvert, 2001a; Driver and Noesselt, 2008; Ho et al., 2009) increased in the past decade and demonstrated that multisensory integration provided clear advantages in terms of subject survival. In other words, estimates from different modalities were more reliable than isolated estimates and contributed to form more meaningful representations of the environment. Behaviorally, integrating information across sensory modalities improved the speed of detection and reduced perceptual ambiguity (Stein et al., 1996; Rowland et al., 2007; Wallace and Stein, 2007). Interestingly, some authors claimed that multisensory integration was an acquired ability (McIntosh et al., 1998; Stein and Stanford, 2008; Brandwein et al., 2011). Electrophysiologically, multisensory processing was described through bimodal neurons activities (Allman et al., 2009) and those were reported almost exclusively in studies of the superior colliculus (SC; Stein and Meredith, 1993). Other studies confirmed similar findings in the cortex (Wallace et al., 1992; Martuzzi et al., 2007) and especially in the auditory cortex of non-human (Kayser et al., 2009; Falchier et al., 2010) and human primates (Foxe et al., 2002), and also in the visual cortex in non-human (Rockland and Ojima, 2003; Wang et al., 2008) and human primates (Giard and Peronnet, 1999; Calvert et al., 2001b). Very few data were available in the motor cortex and less in the premotor cortex (PM; corresponding to Brodmann’s area 6 or M2) yet considered as another candidate for polymodal integration because of the convergence of visual, auditory, and somotosensory inputs (Graziano, 2001). It is this area that we decided to investigate in the present study while presenting auditory (A) stimuli, visual (V) stimuli, or a combination of both modalities (visuo-acoustic, VA) in a non-human primate model, in the context of a detection task.

Behaviorally speaking, two adult macaque monkeys were trained to detect A, V, or VA stimuli and respond to them with a stereotyped reaching arm movement. This detection task was comparable to previous studies conducted in human subjects (Giard and Peronnet, 1999; Molholm et al., 2002; Gori et al., 2011) and in macaques (Cappe et al., 2010). However, for the first time stimuli levels used with monkeys were based on individually determined perceptual thresholds in order to precisely determine the auditory versus visual stimuli intensities required to modify the multisensory gain in motor response latencies. Performances were assessed by measuring the reaction times (RTs), calculating the percentage of correct responses and reporting the errors progression. As expected, RTs were shorter when movements were triggered by cross-modal stimulation than by unimodal stimulation. This behavioral facilitation usually known as redundant signals effect (RSE) might be tested through two different models: (1) The race model (Raab, 1962) which assumed that shorter RTs in a multisensory context were due to the sensory modality which first detected the cue; and (2) the co-activation model (Miller, 1982) which explained that a parallel processing of unimodal channels interacted somewhere in the sensory information processing system.

Electrophysiologically speaking, single units were recorded from a chronic recording chamber anchored above the PM in the same two monkeys executing the behavioral paradigm. The aim was to characterize bimodal neurons in PM possibly responsible for rapid audio-visual integration leading to a fast motor response. This hypothesis was built on the basis of previous studies (Graziano et al., 1997) which reported that aside from a majority of neurons from ventral PM responding to tactile stimuli about 40% were active in response to visual stimuli. Graziano et al. (1999) characterized trimodal neurons too which were active when a supplementary acoustic stimulation was delivered. In addition studies in the dorsal PM from Weinrich and Wise (1982) and Weinrich et al. (1984) demonstrated that some neurons modulated their discharge rates during a visual stimulation. Therefore it became clear that PM which played an important role in the preparation and control of voluntary movements (e.g., Wise and Kurata, 1989; Wise et al., 1997; Luppino and Rizzolatti, 2000) had to be investigated in the context of multisensory integration in order to characterize its contribution to generate a unified percept used to generate quick behavioral responses Therefore the present study is an attempt to link single-unit activities in PM to behavioral performance in order to better understand the neural representations that guide motor behavior.

## Materials and Methods

### Subjects

Two adult non-human primates (*Macaca fascicularis*; Mk-LI and Mk-JZ) were enrolled in the present study. Mk-LI was 9 years old and Mk-JZ was 7 years old at the time of the onset of electrophysiological recordings. The monkeys Mk-LI and Mk-JZ are distinct from the two monkeys used in a previous study from this laboratory based on an earlier, less elaborate version of the psychophysical paradigm (Cappe et al., 2010), with some important differences as outlined in the discussion. The monkeys’ weight was monitored daily and both weighed around 8 kg. When a 10% loss of weight was measured, experiments were interrupted until they recovered their previous weight. Such event did not occur in the course of the present study. Between experimental sessions, the animals shared with other monkeys (groups of two to five animals) a detention room of 45 m^3^ (15 m^3^ until 2010; see e.g., Kaeser et al., 2011; Schmidlin et al., 2011), in which they could freely move and had free access to water. They were never deprived of food but the daily intake was adjusted to the performance in order to not loose motivation. The days without tests, the animals were fed by the animals’ caretakers. Otoscopic examination was carried out regularly to verify that the external ear canal and the tympanic membrane were intact and free of infection. The experiments were conducted according to both guidelines of the National Institute of Health: *Guide for the Care and Use of laboratory Animals* (1996), and of the European Community: *Guidelines for Animals Protection and Use for Experimentation*. Furthermore, the cantonal and federal Swiss veterinary authorities approved the experimental procedures (veterinary authorizations 173-06, 173-07, 156-06, 156-08, 18/10).

### Stimuli

The subjects were trained to perform a detection sensory-motor task with visual and auditory stimuli delivered individually or simultaneously. The tests were carried out in a modified double-walled electrically shielded sound-proof room (compact model, type AB200, Eckel Industries of Canada).

Sounds were delivered under free-field conditions through two loudspeakers (RTO, Hi-Fi 2 Way Speaker System, model HF-10) positioned at equal distance of the monkeys’ heads (∼20 cm), on the left and on the right, and at the same height. The auditory stimulus was a white-noise burst of 250 ms duration generated digitally by RPvdsEx software (Tucker-Davis Technologies System 3, USA) and later converted to analog format by a real-time processor (RP2.1 or RX6, Tucker-Davis Technologies, USA). The calibration of the system was performed with a sound level meter (Brüel and Kjaer, 2231) using a microphone (Brüel and Kjaer, 4189, pre-polarized, 1/2′′) placed at the normal location of the center of the head. Calibration was based on a calculated reference voltage generated at 94 dB SPL at 1 kHz.

Visual stimuli were delivered by a green light-emitting diode (LED 1.9 mm in diameter, Kingbright) positioned in front of the subject, at eyes’ height and at a distance of 23.5 cm. The diode was on during 250 ms and the apparent intensity was digitally controlled through RPvdsEx software (Tucker-Davis Technologies System 3, USA) which supplied current pulses of specific frequencies. The conversion into analog format was provided by a real-time processor (RX6, Tucker-Davis Technologies, USA). This visual stimulation appeared on a black background screen displaying repeatedly (every trial) a white round centered target (2 cm in diameter) to lock the gaze during the experiments. The calibration of the system was performed with a CCD camera (Prosilica ccd camera, Prosilica GE) placed at the normal location of the eyes and expressed in Lux (lx) which corresponds to the luminous flow received by unit area. This calibrated value was related to the current pulses frequency used for supplying the diode therefore allowing us for the rest of the paper to refer to Hertz units instead of Lux.

### Sensory-motor task

**Figure 1:**
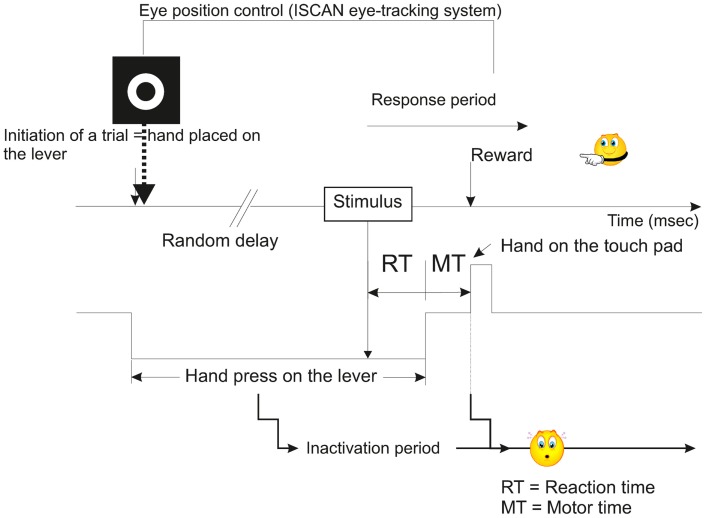
**Schematic representation illustrating the temporal course of a typical trial of the multisensory and sensorimotor integration task used in the present study (modified from Smith and Olszyk, 1997)**. See also Movie S1 in Supplementary Material.

During a first phase of training, which lasted several months (approximately 8–10 months), the subjects were taught a sensory-motor task where they had to release a lever, then press a touch pad in response to either visual (V), or acoustic (A), or VA stimuli. This training was based on a positive reinforcement protocol described previously (Durif et al., 2003; Cappe et al., 2010) and modified from Smith and Olszyk (1997) (Figure 1). Briefly all the recording sessions were performed in a sound-attenuated chamber and the trials were initiated by the animal when pressing a lever with the left hand. At this step, a target was displayed on the screen facing the animal, and although the head was unrestrained, gaze fixation was encouraged to be maintained up to the end of the motor period (in anticipation to electrophysiological recordings where neurons may be responding to gaze direction too). Following this initiation time, a random delay was set ranging from 1 to 4 s in order to minimize anticipation of stimulus onset. The delay ended with the presentation of a stimulus (visual, acoustic, or VA) and the subject was requested to touch a pad positioned above the lever with the left hand. A correct response was rewarded by one banana-flavored pellet (Dustless Precision Pellets^®^ Primate, Grain-Based, Bio-Serv, NJ, USA). If the subject released the lever in absence of stimulus or in anticipation (RT <150 ms), corresponding to a false alarm, a 3-s time out was generated during which it was impossible for the subject to initiate a new trial, and of course to receive a reward. When the motor response occurred after the stimulus with a RT larger than 800 ms, then the trial was considered as erroneous (lack of detection, as in absence of motor response to a true stimulus). The behavioral task was entirely controlled and monitored using a customized workstation, elaborated from RPvdsEx software (Tucker-Davis Technologies, USA) and running on real-time processor devices (RP2.1 or RX6, Tucker-Davis Technologies, USA). The eye-tracking system (ISCAN Inc., USA) was also incorporated into our workstation.

### Thresholds assessment

Both auditory and visual thresholds were obtained by using an adaptive staircase method. This well-known psychophysical technique (Levitt, 1971) is a variation of the method of limits involving both ascending and descending limits and where the test is continued until several reversals are accomplished. Usually threshold is the average of at least six or eight reversal points. The configuration used in the present study was (1) an initial stimulus level having a high probability of a positive response, (2) a decrease of the stimulus level by half after a positive response (or an increase by half after a negative response) therefore the significant initial step chosen (Table 1) and, (3) an average of the last eight reversals. Those steps of the protocol are illustrated in Movie S1 in Supplementary Material (http://www.unifr.ch/neuro/rouiller/research/gerardmat1.php) where the monkey is handling a lever only (training paradigm). For the experiments, the minimum step size was fixed at 2 dB SPL for acoustic stimuli and 2 Hz for visual stimuli (diode pulse rate frequency) in order to not produce thresholds under the precision reached by the calibration of our equipment (for loudspeakers as well as LEDs). Common reported errors of habituation and anticipation have been respectively minimized thanks to (1) a random delay before stimulus onset and (2) a 3-s time out plus no reward whenever the subject prematurely reported detecting the stimulus before its occurrence.

**Table 1:** **Parameters used according to the type of threshold search procedure**.

	Visual	Acoustic
Initial intensity	1000 Hz (79 dB)	50 dB SPL
Steps	100 Hz (33 dB)	10 dB SPL
Step division	Division by 2 after each inversion	
Min. step	2 Hz (−45 dB)	2 dB SPL
Inversions	13 Inversions	
Threshold	Last four peaks and four valleys average	

Both auditory (binaural) and visual thresholds were expressed in decibels. Therefore visual thresholds had to be converted from Hertz into decibels according to the formula: $\mathrm{dB = 20lo}g_{\mathrm{10}}\left(\frac{P_{2}}{P_{1}}\right)$ where *P*_1_ = 18.98 Hz (lowest visual level detected by 10 healthy human subjects from our laboratory) and *P*_2_ the tested level.

The threshold testing was completely automatized with RPvdsEx environment (Tucker-Davis Technologies, USA) and with several subroutines elaborated with MATLAB^®^(The Mathworks, Inc., USA) and Labview (National Instruments™, USA).

### Multisensory sessions

For multisensory sessions, the sensory-motor task was similar to the one developed for determining thresholds. The gaze was still locked but in addition the eye position was monitored using an ISCAN eye-tracking system (RK-426 Pupil/Cornal Reflection Tracking System, ISCAN^®^, Inc., USA) to ensure that the subject did not move his eyes from the beginning of the trial till the detection of the stimulus. During this cross-modal task, auditory and visual stimuli were presented individually or in combination (see Movie S1 in Supplementary Material). The random distribution of these three conditions was provided by the customized workstation and more specifically by the RPvdsEx software (Tucker-Davis Technologies, USA). Therefore a daily session consisted of at least 200 trials randomly distributed into acoustic, visual, and VA conditions. At these daily sessions, the tested intensities were fixed based on the threshold values obtained previously plus 10 dB. Levels of performance were considered as stable when the daily sessions contained generally less than 15% of erroneous trials (false detection or anticipation of motor response). At that step, a head fixation device was anchored to the skull (see below). Monkeys were then re-trained over a period of 1 month to execute the entire auditory-visual-motor task with the head restrained in order to re-establish a stable level of performance. The recordings (psychophysics) went on afterward over several months followed finally by electrophysiological investigations. The daily recording sessions generated data such as RTs and percentage of correct performance with identification of different errors, such as execution or detection errors.

### Surgery

As mentioned above, when the subjects reached a daily stable level of performance, a first surgery aimed at implanting a head fixation device (for details about the methods and the device see (Lanz et al., 2013). Briefly, this device was used to restrain the monkey’s head movements in order to allow eye position monitoring and therefore was anchored to the skull. No dental or orthopedic cement was used but only screws and an osseous integration facilitation procedure. A second surgery was performed after several months in order to implant a chronic recording chamber in tekapeek, allowing daily single-unit recordings in the PM (again more detail on the method and the device can be found in Lanz et al., 2013).

### Electrophysiology

Electrophysiological recording sessions were performed when the monkeys were engaged in the protocol of multisensory-motor detection task and exhibited a stable level of behavioral performance (see details in the Section “Multisensory Sessions” above). Neuronal extracellular activities were recorded with tungsten microelectrodes (5–7 MΩ impedance from FHC, Bowdoinham, ME, USA), advanced perpendicularly with respect to the dura through the chronic recording chamber into the PM. The electrode driving system used was from Narishige^®^(Narishige International limited, Japan). For both monkeys, the activities recorded were derived from single neurons in the right PM (contralateral to the arm used to execute the motor task). At the exploratory stage, auditory and visual stimuli were fixed at 30 and 90 dB above threshold, respectively, and when a putative interesting neuron was identified (stable activity over approximately 100 trials), a new acquisition was started again with stimuli at different levels of lower intensities. Recording sessions took place on a daily basis during a period of several months (2–3 months) and concurrently behavioral data were stored (as described in the Section “Multisensory Sessions” section). Neurons were discriminated using the principal component feature space spike sorting software (SpikePac from Tucker-Davis Technologies, USA) which allowed us to select and sort out data during acquisition but also to perform playback of stored data for dynamic visualization of neural activities. These data were then exported into MATLAB^®^(The Mathworks, Inc., USA) to perform off-line analysis. A subroutine was designed in order to sort out data into three matrices (A, V, and VA) and to build-up dot rasters and peri-stimulus time histograms (PSTHs) where every trial’s activity is aligned against the onset of the stimulation. Neuronal responses were transformed into spikes per second. For the analysis, three periods of the same duration (200 ms) were defined: a reference period of “spontaneous” activity before delivery of the stimuli, an activity period during the presentation of the stimuli (A or V or VA), and a post-stimulation period following the latter. The neuron baseline activity was defined as the mean of activities recorded during the reference period. For each condition (A, V, or VA), comparisons were carried out between the discharge means of the reference period and the activity period and, between the reference period and the post-stimulation period with a two-sample *t*-test. Another comparison with the same test was performed between activity periods of different conditions and between post-stimulation periods. A significant response of the neuron was identified when *p* < 0.05. A standard deviation (SD) was calculated and helped us to visually assess when the evoked activity was overshooting the mean baseline activity plus 2 SDs (excitation; see e.g., Wannier et al., 2002; Durif et al., 2003) or was below the mean baseline activity minus 1 SD (inhibition).

## Results

### Absolute sensory thresholds

Auditory and visual behavioral thresholds were first evaluated independently in our two subjects (Mk-LI and Mk-JZ) with psychophysical tools (adaptive staircase method) using respectively white-noise bursts or focused flashing lights. Mean thresholds and SDs were calculated from 15 sessions over 5 months of training (Mk-LI) and 2 months of training (Mk-JZ) for each sensory modality. Performing a Mann–Whitney test confirmed that no significant difference was observed between the two animals for visual thresholds (*p* = 0.06, mean threshold = 24.6 dB SPL in Mk-LI and 23.6 dB SPL in Mk-JZ). However a difference for auditory thresholds was observed (*p* = 0.04, mean threshold = 7.8 dB in Mk-LI and 6.6 dB in Mk-JZ).

### Reaction times: mean values and time courses

**Figure 2:**
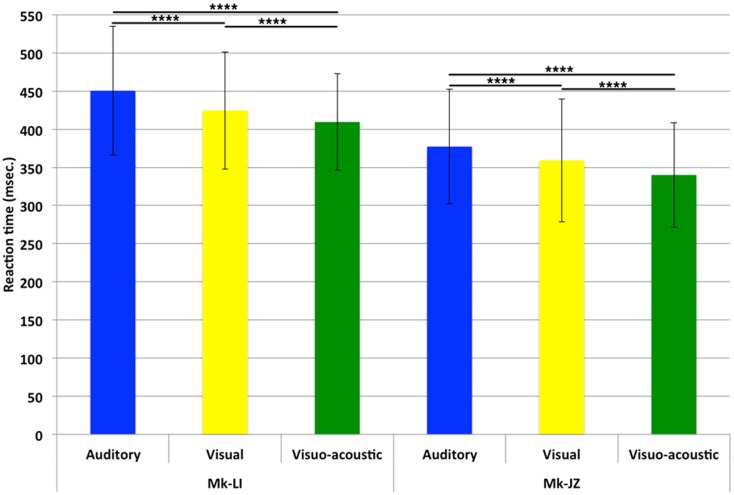
**Mean reaction times and their SDs obtained at 10 dB above unisensory thresholds**. Data derived from several months of daily sessions in Mk-LI (12 months) and in Mk-JZ (10 months). ****p < 0.0001 (Mann–Whitney test).

For both monkeys, following the initial training, a period of stable behavioral performance was selected and data were extracted from sessions comprising at least 200 trials. Data were usually collected daily, 5 days per week. The distribution of RTs in response to visual, auditory or VA stimulations is displayed in Figure 2. These data were obtained over a period of respectively 12 (Mk-JZ) and 10 (Mk-LI) months before the initiation of the electrophysiological recording period. All data were obtained at 10 dB above sensory thresholds (see paragraph above), corresponding to 17 dB SPL for the auditory stimulation and 34 dB (representing 950 Hz in diode current pulses) for the visual stimulation. The results showed that mean auditory RTs [450 ms (*n* = 1,391) for Mk-LI and 377 ms (*n* = 1,091) for Mk-JZ] were significantly different (Mann–Whitney test, *p* < 0.0001) from visual RTs [424 ms (*n* = 1,648) for Mk-LI and 359 ms (*n* = 1,292) for Mk-JZ]. VA RTs [409 ms (*n* = 1,883) for Mk-LI and 340 ms (*n* = 1,091) for Mk-JZ] were significantly shorter than auditory RTs (*p* < 0.0001) and visual RTs (*p* < 0.0001; Figure 2) in both monkeys. Mean auditory RTs were about 20 ms longer than visual RTs. RTs resulting from VA stimulation were shorter than the fastest unimodal stimulation (visual in the present case). In addition, subject Mk-JZ showed clearly shorter RTs (approximately 70 ms less) than subject Mk-LI.

**Figure 3:**
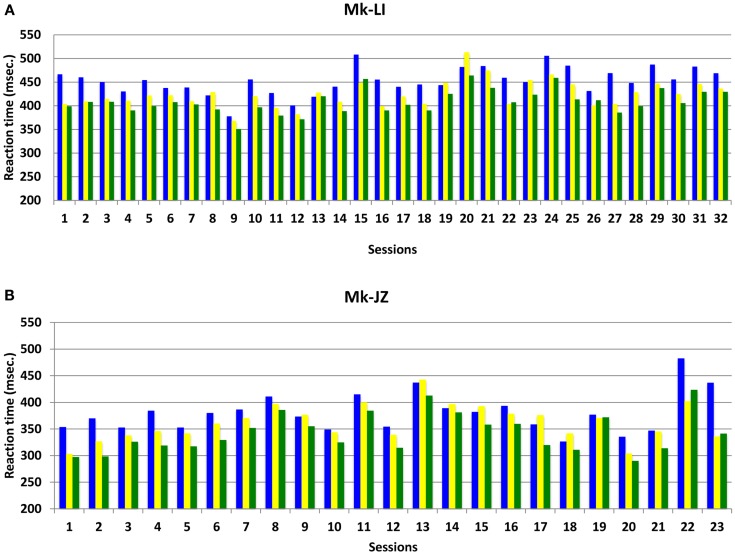
**Mean reaction time progression over time (daily sessions) for subjects Mk-LI (A) and Mk-JZ (B) with intensities 10 dB above unisensory thresholds**. The data were collected from a restricted time window (32 daily sessions in Mk-LI and 23 daily sessions in Mk-JZ), preceding the electrophysiological data collection. RTs in response to auditory stimuli are in blue, to visual stimuli in yellow and to both modalities delivered simultaneously in green.

The progression and variability of mean RTs with time for both subjects Mk-LI and Mk-JZ are depicted in Figure 3, during the time window preceding the electrophysiological recording. In this Figure, data from Mk-LI were collected over 32 sessions and showed in most cases the same general pattern as described in Figure 2, namely mean VA RTs shorter than mean visual RTs, themselves shorter than mean auditory RTs. In Figure 3, data from Mk-JZ were obtained over 23 sessions and showed the same pattern. In both monkeys, the RT values were considered as stable enough to initiate the electrophysiological recordings.

### Time course of errors

**Figure 4:**
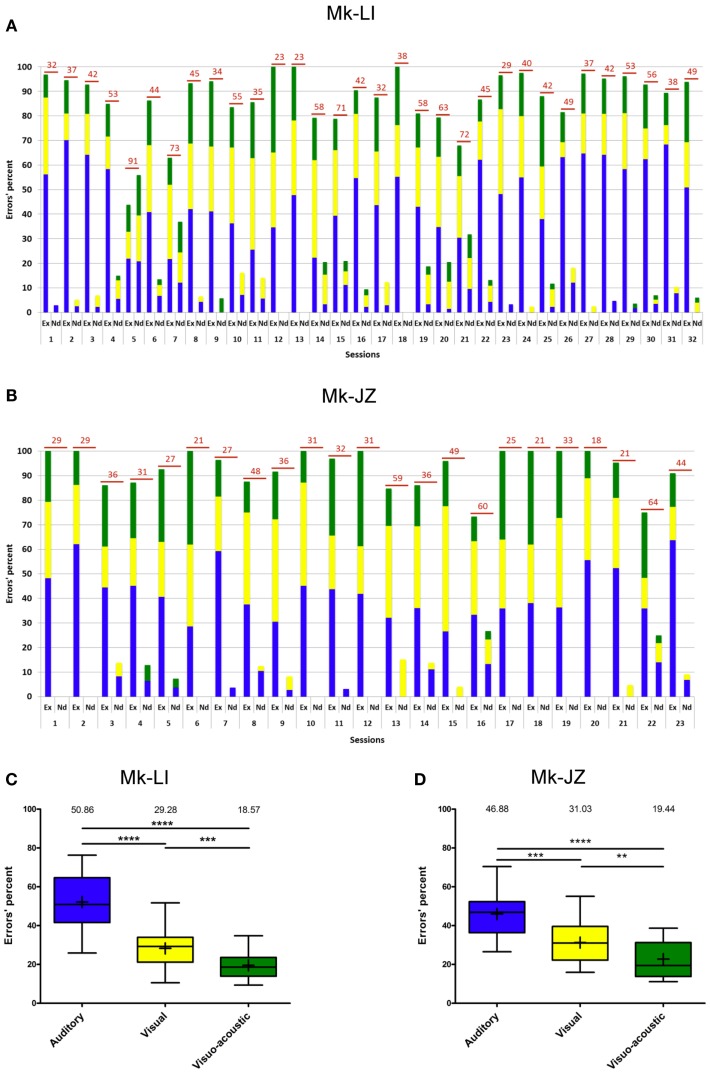
**Errors’ progression for both subjects (same time window as in Figure 3)**. **(A)** for Mk-LI and **(B)** for Mk-JZ. The total number of errors per session was indicated on top of each bars. “Ex” stood for Execution errors; “Nd” for Not detected errors. The percentages of errors displayed in **(A,B)** were distributed in the form of box and whisker plots **(C,D)**. The end of the whiskers represented maxima and minima. The Mann–Whitney test showed *p*-values results as ** when *p* < 0.01, *** when *p* < 0.001 and **** when *p* < 0.0001.

During the time window of psychophysical data collection, the proportion of erroneous trials was generally below 15%. Errors recorded during the sensory-motor detection task are reported in Figure 4A for subject Mk-LI and Figure 4B for subject Mk-JZ, respectively). Errors have been divided into “execution” errors when the animal was deviating from the protocol and “not detected” errors when an expected response to a stimulus did not happen. The distribution over time and the color code for errors occurring during visual, auditory or VA stimulation was the same as the one used in the previous section. As far as the “execution” errors were concerned for subject Mk-LI (Figure 4A), differences were significant across the sessions and within the three sensory modalities [*F*(2, 93) = 79.35, *p* < 0.001]. The “not detected” errors also showed significant differences across the sessions and within the three sensory modalities [*F*(2, 93) = 3.26, *p* = 0.04], whenever they were all three available.

In Figure 4B, the “execution” errors made by subject Mk-JZ showed significant differences across the sessions and within the three sensory modalities [*F*(2, 66) = 25.0, *p* < 0.001]. The “not detected” errors also showed significant differences across the sessions and within the three sensory modalities [*F*(2, 66) = 3.61, *p* = 0.03]. From a general point of view, both graphs (Figures 4A,B) showed that the two subjects did mainly execution errors rather than detection errors and that the errors were randomly distributed over time. As shown in Figures 4C,D (*n* = 32 and 23 respectively), and as expected for multisensory facilitation, the percentage of errors was lowest in the VA condition, as compared to the A and V unisensory conditions. Furthermore, there were fewer errors in the V condition than in the A condition.

### Stimuli intensity effects

**Figure 5:**
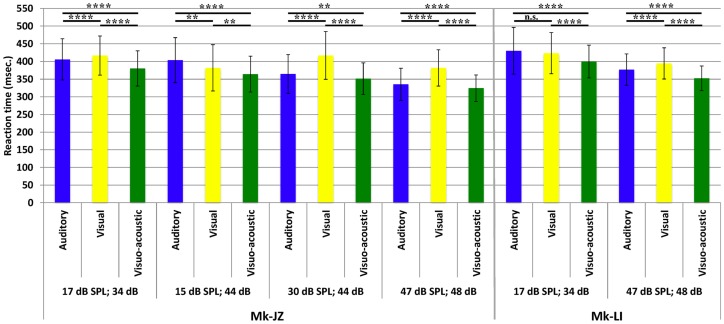
**Mean reaction times (with their SDs) obtained for different combinations of supra-threshold intensities and for both monkeys (Mk-JZ and Mk-LI)**. n.s.: *p* ≥ 0.05; **p < 0.01; ****p < 0.0001 (Mann–Whitney test). The data were derived from the electrophysiological daily sessions’ collection.

The variations of RTs as a function of stimuli intensities are reported in Figure 5. The first pool of data (*n* ≈ 1,000) collected for each animal was obtained at 10 dB above unisensory thresholds which corresponded to 17 dB SPL for auditory stimuli and 34 dB for visual stimuli. In Mk-JZ, three other supra-threshold auditory intensities have been tested (15, 30, and 47 dB SPL) combined with 44 and 48 dB for visual stimuli. The differences between uni- versus multi-sensory RTs for each subject have been tested with a non-parametric *t*-test (Mann–Whitney test) and were significantly different between all stimulation conditions (*p* < 0.0001). In Mk-LI, two supra-threshold intensity conditions are shown (Figure 5), also exhibiting statistically significant RTs differences, except for A versus V at A = 17 dB SPL and V = 34 dB. As a second step, the effect of stimulus intensity on RTs has been tested for each sensory modality through a one-way ANOVA for Mk-JZ and a *t*-test for Mk-LI. It resulted that an increase of the auditory intensity decreased significantly the response time (*p* < 0.0001) but not between 15 and 17 dB (not significant); an increase of the visual intensity decreased significantly the RT between 34 and 48 dB (*p* < 0.0001), as well as between 44 and 48 dB (*p* < 0.0001). However no difference was demonstrated between 34 and 44 dB. Concerning the combined stimuli, we obtained a significant difference (*P* < 0.0001 and *P* < 0.01 between VA = 15 dB SPL; 44 dB and VA = 30 dB SPL; 44 dB, which demonstrated that RTs decreased when intensities of stimuli increased. For Mk-LI the increase of intensity decreased significantly the RT (*P* < 0.001) for all conditions (A, V, and VA).

To determine if the RSE are consistent with the race model or the co-activation model, we applied for every parameter a cumulative distribution function and a Miller’s race model inequality (Miller, 1982), respectively. The purpose of this equation is to test whether the probability for a RT during a cross-modal stimulation is higher than the summation of both uni-sensory RT.

**Figure 6:**
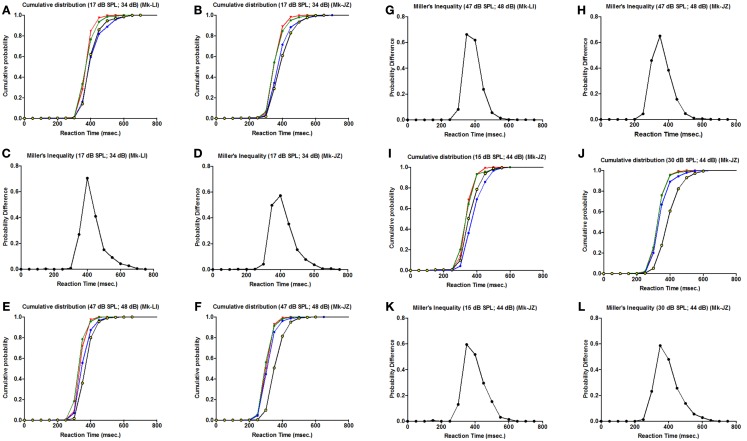
**Cumulative distribution function for different intensities for both monkeys (Mk-LI and Mk-JZ)**. **(A,B,E,F,I,J)** are for the cumulative distribution functions for Mk-LI **(A,E)** and for Mk-JZ **(B,F,I,J)**. **(C,D,G,H,K,L)** are for the Miller’s Inequality for Mk-LI **(C,G)** and for Mk-JZ **(D,H,K,L)**. In the cumulative distribution graphics, the blue curve represent the auditory condition, the yellow/black curve the visual condition and the green curve the visuo-acoustic condition. The red curve is the model predicted by Miller’s race model inequality (Miller, 1982).

As a first step, the cumulative distributions are displayed in Figures 6A,B,E,F,I,J, respectively for each monkey and each parameter tested (as in Figure 5). The more the curve is shifted to the left, the shorter is the RT, which means that we have a higher probability at a given RT latency. For all parameters, there is a leftward shift for cross-modal stimulation (AV) (corresponding to green curves in Figures 6A,B,E,F,I,J), as compared to the A or V curves.

In complement, an analysis of the inequality of Miller was tested for every parameter (Figures 6C,D,G,H,K,L). This inequality is defined as: *P*[RT(VA)] < [*P*(RT(A)] + *P*[RT(V)] – [*P*(RT(A)] × *P*[RT(V)] where *P*(RT) is the cumulative probability density function of RT. This standard analysis (see Murray et al., 2005) consisted in calculating the probability distribution for each condition minus their joint probability. This model represented the upper limit that would be explainable by probability summation. In the panels quoted above, we could observe violations of the race model (i.e., positive values for the probability difference) for all parameters tested, which invoked neural response interactions. Furthermore, we could notice a decrease of the probability differences’ values when the intensities increased (in Figures 6G,K,L).

### Bimodal gain

**Figure 7:**
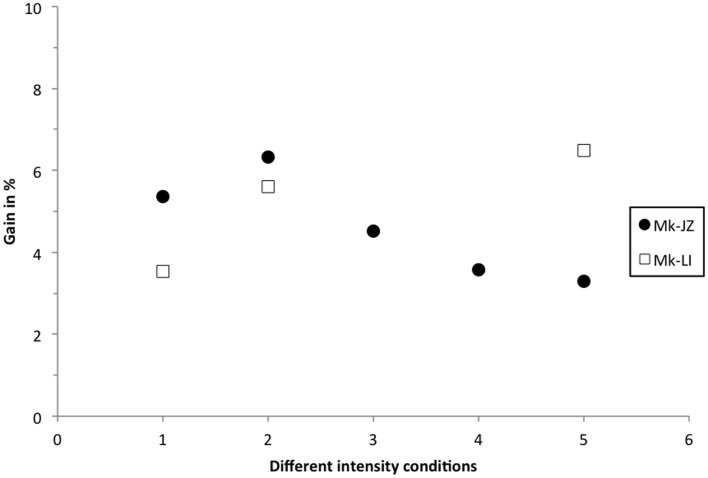
**Multisensory gain expressed in percent of the mean RT in visuo-acoustic conditions as compared with the mean RT of the fastest unisensory condition**. Filled circles display the values for Mk-JZ as a function of the stimulus conditions shown in Figure 2 [Condition 1 = 17 dB SPL (auditory), 34 dB (visual)] and Figure 5 [Condition 2 = 17 dB SPL (auditory), 34 dB (visual)]; Condition 3 = 15 dB SPL (auditory), 44 dB (visual); Condition 4 = 30 dB SPL (auditory), 44 dB (visual); Condition 5 = 47 dB SPL (auditory), 48 dB (visual)]. Opened squares display the values for Mk-LI as a function of the same stimulus conditions.

Similarly to the calculation reported by Stein and Meredith (1993), the multisensory gain was plotted in Figure 7, corresponding to the decrease of the mean RT in percent in the VA condition, as compared to the faster mean RT in unisensory conditions. At 10 dB above threshold, subject Mk-JZ showed a bimodal gain ranging from 5 to 6% whereas, in subject Mk-LI, the bimodal gain ranged from 3.5 to 6%. At intensities higher than 10 dB above thresholds (conditions 3–5 in Figure 7), a lower gain was observed than at 10 dB above threshold for Mk-JZ (ranged from 4.5 to 3%) but not for Mk-LI exhibiting a gain of 6.5% at high intensities. More generally, the maximal gains were around 5 to 6% and the principle of inverse effectiveness was largely verified in Mk-JZ, but less so in Mk-LI (Figure 7).

### Electrophysiological results

The goal here was to test the hypothesis that the sensory modality influences the coding of the motor response during its preparation and planning within and/or after the RT period.

**Figure 8:**
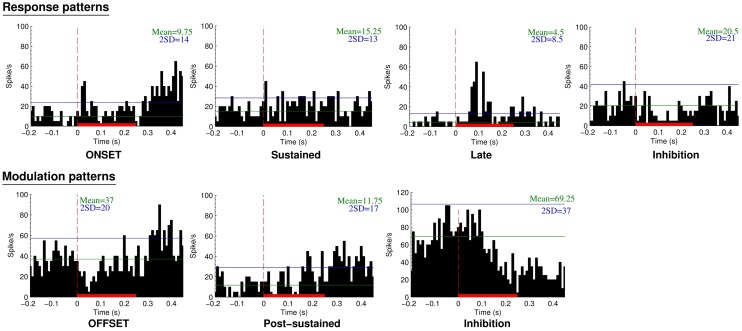
**Neuronal categories obtained from recordings in PM during supra-threshold auditory, visual, or visuo-acoustic stimulations**. The top panel considers the responses during the stimulation period whereas the bottom panel is based on the modulation patterns during the post-stimulation period. The peristimulus histograms (PSTH) display the number of spikes over 40 trials as a function of time before, during (red solid bar), and after the presentation of the stimulus. The vertical red line at time zero is the onset of stimulus presentation. Bin width is 10 ms. The horizontal green line represents the mean discharge rate of reference derived from the period preceding the presentation of the stimulus. The blue horizontal line is the mean discharge rate of reference plus 2 SDs, considered as the limit of confidence for an excitatory response.

A total of 132 neurons, pooled between Mk-JZ and Mk-LI, were recorded from the PM while the subjects were performing the VA detection task. Electrodes tracks were directed toward PM, mainly its dorsal division (PMd) and were distant from each other by 0.5 mm along the rostrocaudal and mediolateral axes. A surface map of the electrodes coordinates was constructed for each monkey. Figure 8 shows a representative set of PM neurons and categories were defined according to the discharge patterns. The top panel entitled “Response patterns” corresponded to responses obtained during the period of stimulation (250 ms duration). All the responses of these types were significantly different from the 200 ms-reference period according to the two-sample *t*-tests performed for each single unit. The four types of response patterns were:

– Onset: when a strong and sharp excitation happened after the onset of the stimulus. It corresponded visually to an overshoot over the average activity of reference plus 2 SDs,
– Sustained: when an enhanced activity was observed all along the stimulation period,
– Late: when a strong and sharp excitation arose on the second half of the stimulation period (∼100 ms and later).
– Inhibition: when a significant decrease of the activity happened during the stimulus, corresponding visually to a decrease of activity below 1 SD subtracted from the average activity of reference.

The bottom panel entitled “Modulation patterns” considered significant variations of neuronal activity with respect to the period of reference observed during the post-stimulation period. The two-sample *t*-tests performed over a period of 200 ms were significant against the reference period. In this category three types of responses were identified:

– Offset: when a short period of activity happened immediately after the end of the stimulus,
– Post-sustained: when an enhanced activity was observed all along the post-stimulus period,
– Inhibition: when a decrease of activity was observed after the stimulation period.

In addition to this categorization we determined the percentage per modality of the neurons expressing a significant change in their activity during or after the period of stimulation (Table 2). One could notice that the percentages of modulated neurons (change of activity post-stimuli) were roughly two times higher than the number of neurons responding during the stimuli. These percentages were stable across sensory modalities. Concerning the stimulus responding neurons the table shows a slightly higher but not significant (χ^2^ = 3.29; df = 2; *p* = 0.19) number of multisensory neurons than unisensory neurons.

**Table 2:** **Proportion of neurons exhibiting a change in their activity as a function of the sensory modality and the epoch**.

	Acoustic		Visual		Visuo-acoustic	
	Stimulus	Post-stimulus	Stimulus	Post-stimulus	Stimulus	Post-stimulus
Percent	25 (*n* = 33)	60.6 (*n* = 80)	21.2 (*n* = 28)	57.6 (*n* = 76)	34.1 (*n* = 45)	56.1 (*n* = 74)

**Figure 9:**
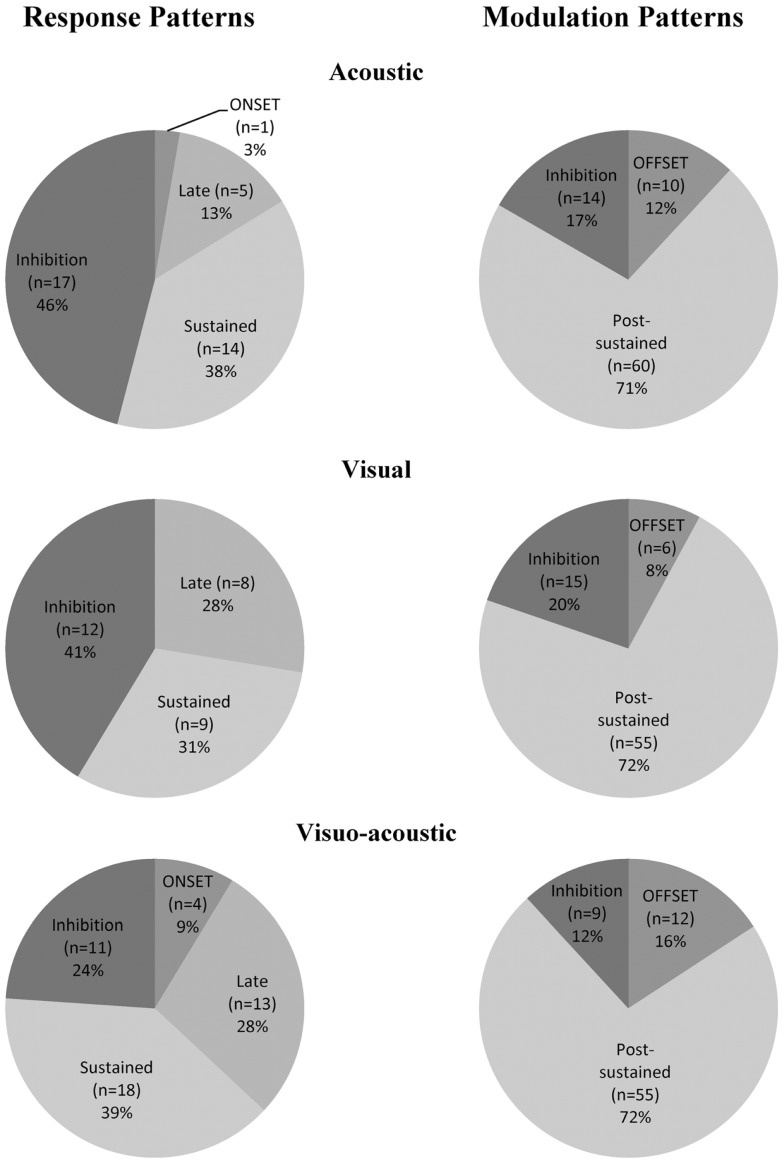
**Proportion of acoustic-, visual-, and visuo-acoustic- responding cells from PM according to the categorization presented in Figure 8**. Numbers represent percentages over the entire cell population from the two monkeys together. Movie S1 in Supplementary Material: illustration of the sensory-motor detection task execution.

Considering further the pattern of activity presented above (Figure 8), proportions of unimodal and multimodal neurons in PM are depicted in Figure 9. The indicated percentages resulted from a calculation against the total number of neurons expressing an activity within a modality and a pattern. The main activity enhancements corresponded to the “sustained” (35%) and “post-sustained” (72%) types whereas, the inhibition (between 12 and 46%) represented a minority among the recordings. However the inhibition was more frequent in response patterns compared to modulation patterns. One could also note that the combined modality expressed the least percentage of inhibition, especially for response patterns where it was statistically significant: χ^2^ = 7.19; df = 2; *p* = 0.03. In general, this side of Figure 9 showed significant differences in the distribution (χ^2^ = 25.27; df = 6; *p* = 0.0003). By contrast, modulation pattern distributions were comparable across the three modalities as shown by a χ^2^ = 4.68; df = 4 and *p* = 0.32 (no significant differences detected).

It is noteworthy that a neuron could express several patterns during and after the stimulation. For example, a neuron responding to an auditory stimulus could present a “Response pattern” formed by an “Onset” followed by a “sustained” activity or, a “Modulation pattern” formed by an “Offset” followed by a “post-sustained” activity. Such neurons were reported in Table 3 and grouped per sensory modality.

**Table 3:** **Proportion of neurons expressing two different patterns per sensory modality**.

	Acoustic	Visual	Visuo-acoustic
Percent	18.8 (*n* = 18)	17.6 (*n* = 16)	27.7 (*n* = 26)

## Discussion

As compared to a previous study from this laboratory (Cappe et al., 2010) based on two monkeys, the present report extends to two more macaque monkeys the observation that RTs in response to combined acoustic and visual stimuli are significantly shorter than RTs in response to separate, individual presentation of acoustic or visual stimuli (Figures 2 and 5). In line with the study of Cappe et al. (2010), the bimodal gain (RT decrease) expressed in percent of the RT obtained in response to unimodal stimulation is in the order of 5% (Figure 7). The present study demonstrates that the bimodal gain (reduction of RTs in the VA condition) is robust, as it was maintained in spite of several months of training, followed by a time window of several months during which the psychophysical data were collected daily (Figure 2), as well as during a subsequent short period of acquisition preceding the electrophysiological investigations (Figure 3). The significant decrease of RTs in response to bimodal stimulation (as compared to the shorter unimodal RTs) was observed for a range of intensities going from 10 dB above threshold up to about 40 dB above threshold, whereas in the study of Cappe et al. (2010) this bimodal effect disappeared mostly at 30 dB above the acoustic threshold, as well as for higher intensities. The loss of bimodal facilitation identified by RTs decrease at strong intensities is in line with the well-established principle of inverse effectiveness (Stein and Meredith, 1993; Holmes, 2009). For this reason, intensities higher than 40 dB above acoustic or visual thresholds were not considered in the present psychophysical study. A parallel between the principle of inverse effectiveness observed at behavioral level (see e.g., Mk-JZ in Figure 7) and the activity of single neurons is not straightforward. Indeed, the absolute behavioral threshold measured for a given sensory modality is not directly linked to the threshold of individual neurons, as reflected by its discharge rate. For this reason, in our electrophysiological investigations in PM, it was necessary to use in most cases stimuli intensities higher than the behavioral thresholds, in order to detect an influence of the stimulation paradigm on the neuronal activity.

The design of the present study exhibits some substantial differences as compared to the experimental paradigm of Cappe et al. (2010). First of all, the latter authors used as visual stimulus a flash light covering spatially a large portion of the monitor facing the subject. In contrast, in the present paradigm, the visual stimulus was restricted to a spatially limited LED, requiring a precise control of gaze toward this visual target. Even more important, the authors (Cappe et al., 2010) acknowledged that the visual thresholds were not accurately determined. As far as the auditory threshold is concerned, in Cappe et al. (2010), it was derived from the behavioral task itself by extrapolating the intensity generating 75% of correct trials. As a result, in one monkey (MK2 in Cappe et al., 2010) the auditory threshold was only approximated, between 10 and 20 dB SPL. The present study is clearly different, with unimodal thresholds precisely determined in dB for each sensory modality, using an automatized paradigm distinct from the multisensory detection task, based on a preceding, systematic, and high resolution adaptive staircase method, implemented here for the first time in macaques. The two monkeys enrolled in the present study exhibited fairly close threshold values, although it was significantly different for the visual threshold, but not the auditory one. The auditory thresholds (6.6 dB SPL in Mk-JZ and 7.8 dB in Mk-LI) observed here are consistent with the thresholds (1–8 dB SPL range) obtained from other macaque species (Stebbins et al., 1966; Stebbins, 1973, 1975; Pfingst et al., 1978; Smith and Olszyk, 1997) with different methods and with the monkey MK1 from Cappe et al. (2010).

A further difference with the study of Cappe et al. (2010), in which the controlled variation of intensities was focused on the acoustic stimulus, the present study tested different intensity levels well defined with respect to both the acoustic threshold and the visual threshold (Figure 5). In the study of Cappe et al. (2010), for acoustic intensities close to threshold (10 dB above), the acoustic RTs were longer than visual RTs, but this was the other way around at all other acoustic intensities tested, with auditory RTs significantly shorter than visual RTs (see Figure 3 in Cappe et al., 2010). In the present study, visual RTs were also shorter than auditory RTs when stimuli were presented at 10 dB above the respective unimodal thresholds (Figure 2 of the present study). At intensities higher than 10 dB above thresholds, the auditory RTs tended to be shorter than visual RTs (Figure 5), but this was less systematic than in Cappe et al. (2010). This slight difference may be explained by the different visual stimulus used or by an inter-individual difference across monkeys. Finally, as an extension of the early study of Cappe et al. (2010), the present study provides quantitative data regarding the occurrence of errors in the behavioral task, with clear demonstration of a reduction of errors in the VA conditions.

Briefly, a comparison with the study of Molholm et al. (2002), conducted in human beings could be made. Indeed, the protocol is also based on a detection sensory-motor task, and not discrimination. However, it is necessary to mention that the auditory and visual intensities were larger than the absolute sensory thresholds. The results in humans also showed RTs facilitation during multimodal stimulations. Nevertheless the differences between the unisensory RTs and the multisensory RTs (between 42 and 50 ms) were larger in humans than in monkeys in our study. In Molholm’s study the violation of the race model demonstrated that the observed facilitatory effect did not result from the auditory and visual neural integrative processes alone.

In a general manner, we observed that RTs were facilitated (shortened) when the two modalities (A and V) were delivered simultaneously, as compared to RTs obtained after unimodal stimulation (A or V). The RSE is demonstrated by the cross-modal RTs exceeding the predictions established by the probability summation. To determine if our RSE are the resultant of the race model or the co-activation model we applied for every condition the Miller’s inequality. Due to the results consistent with a model violation (as shown in Figure 6), we could conclude that, in case of multisensory stimulation, a neural response interaction occurred before the monkey’s movement generation.

Several anatomical studies provide possible mechanisms and locations for early interactions between distinct sensory modalities, as representing putative convergence of information at the origin of these RSE. At cortical level, there are connections between different sensory areas, referred to as heteromodal connections. For example, the parietal VIP area in the monkey receives inputs from the auditory, visual, somatosensory, and vestibular systems, as well as from polysensory areas (Duhamel et al., 1998; Schroeder and Foxe, 2002). Consequently, the neurons of VIP express multimodal responses. Recently it was demonstrated that cortical areas considered as unisensory may have direct connections with other unisensory areas (Schroeder et al., 2001; Cappe et al., 2007; Kayser et al., 2007). In particular, it was demonstrated that visual cortical areas are reciprocally connected with auditory cortical areas (Falchier et al., 2002). These studies are consistent with multimodal interactions which can take place at relatively low levels of the chain of cortical information processing. However, in order to generate very fast motor response to multisensory stimuli (faster than unimodal stimulation), even earlier convergence of sensory information is likely, at subcortical level. For instance, there is evidence for rapid multisensory integration at the level of the superior colliculus (Meredith and Stein, 1986; Cuppini, 2010) a midbrain structure providing access to the motor system (Sparks, 1986; Rezvani and Corneil, 2008; Sommer and Wurtz, 2008). A recent anatomical study (Cappe et al., 2009) also provided indirect evidence in favor of low level, early multisensory integration in the thalamus. Besides its classical role of relaying sensory information to the cerebral cortex with reciprocal modulating feed-back corticothalamic projections, the thalamus is also playing a role in a driving, feed-forward projection system, representing an anatomical support for rapid and secure transthalamic transmission of information from a low level (unimodal) cortical area to another unimodal cortical area (see for review, Rouiller and Welker, 2000; Sherman and Guillery, 2002, 2006; Cappe et al., 2009). These feed-forward transthalamic loops involve corticothalamic projections terminating with giants endings, consistent with fast and secure synaptic transmission, which may favor faster and more reliable motor response to bimodal stimuli, as compared to unimodal ones. As hypothesized by Cappe et al. (2009), the pulvinar nucleus of the thalamus (mainly its medial nucleus PuM) receives projections from different sensory cortical areas, and then is in position to send rapidly the unified multisensory information to the motor system, via its thalamocortical projection to the PM. Furthermore, PuM receives projections from the superior colliculus. These anatomical data need to be confronted with neurophysiological investigations, by means of single neuronal recordings or EEG in animals (Meredith and Stein, 1986; Romanski, 2007; Bizley and King, 2008; Cohen et al., 2011; Perrault et al., 2011), and also by fMRI and EEG in human subjects (Foxe et al., 2000; Molholm et al., 2002; Doehrmann et al., 2010; Senkowski et al., 2011). Some of these functional data showed neuronal activity in cortical areas (auditory, somatosensory, visual area, etc.) in connection with multisensory integration. The present behavioral data and their electrophysiological counterparts in PM represent an attempt along this line to elucidate the pathways involved in early multisensory and sensorimotor integration. Along this line, the same two monkeys (Mk-JZ and Mk-LI), after completion of the neuronal recordings in PM, will be implanted with another chronic recording chamber to permit access to the pulvinar nucleus of the thalamus, in order to test its possible contribution to such a multisensory detection task.

The electrophysiological data in PM (Figures 8 and 9) are in line with previous studies reporting single neurons’ responses to auditory or visual stimuli (Graziano, 1999, 2001; Graziano et al., 1999) in this motor cortical area, in the context of the control of a motor act triggered by a sensory stimulus. However, in previous studies in PM, the sensory stimulus was generally used so that it represents a cue-signal from which the subject has to select an appropriate motor response in a conditional behavioral task with delay, the latter being followed by a go-signal prompting the motor response itself. In particular, variations of the cue-signal within a given modality (different colors, different positions, etc.) instruct distinct motor acts (e.g., movements in different directions). With that respect, the present study is clearly different as two distinct unimodal stimuli triggered the same motor response, without any delay up to a go-signal before the subject has to respond behaviorally to the stimulus. The present paradigm is a simple detection task with fast stereotyped motor response irrespective of the stimulus property, whereas conditional tasks with delay involve a discrimination of different stimuli and their interpretation for conversion into the appropriate motor act among a palette of possible motor responses. To our knowledge, the present study is the first investigation in PM based on such detection behavioral paradigm, comparing unimodal versus bimodal stimulations. Not surprisingly then, the present electrophysiological data derived from PM (Figures 8 and 9) in the context of the detection task appear different as compared to PM data derived from a conditional task with delay in which various visual cue signals (left and/or right positioned LEDs) instructed distinct motor responses in the form of unimanual or bimanual reach and grasp movements (Kermadi et al., 2000). In the latter study, the responses to the cue signals were relatively frequent in PMd (in about 40% of the neurons) and they were strong (see e.g., Figure 5A of Kermadi et al., 2000). In the present study (detection task), the responses to the sensory stimuli in PM are clearly less frequent and not as prominent as those observed by Kermadi et al. (2000). This difference may be interpreted in the sense that in the conditional task with delay, the monkey had to pay more attention to the cue-signal, as it was crucial to select a specific motor response among three possibilities. In the present detection task, the sensory stimulus does not represent a basis to select a motor response, as only one motor act is requested and therefore the pertinence of the sensory stimulus is limited to the time at which the motor act has to take place. For this reason, less prominent and less frequent “sensory” responses in PMd can be expected in the present detection task, as compared to conditional tasks with delay comprising behaviorally more meaningful sensory instructions. Actually, the sensory stimuli delivered in the present detection task are more comparable to the go-signal of the conditional tasks with delay. In the studies of Kermadi et al. (1998, 2000), “sensory” responses to the go-signal were fairly rare (not only in PMd, but also in M1 and SMA), thus in line with the low occurrence of responses to the visual and/or acoustic stimuli in the present detection paradigm.

To some extent, the response types in PM illustrated in Figure 8 in the present detection task of visual and/or auditory stimuli is reminiscent of the responses observed in the ventrolateral prefrontal cortex, when faces and/or vocalization stimuli were presented to awake behaving monkeys (Romanski, 2007; see her Figure 4). However, again, neurons exhibiting multisensory integration were clearly more frequent in the ventrolateral prefrontal cortex (50% of bimodal neurons; Romanski, 2007) than found in PM for the present detection task (34.1%). The difference between prefrontal cortex and PM may be explained by the well-known and prominent role of the former in associating distinct sensory modalities, especially vision and hearing, as well as by substantial differences related to the stimuli properties. In the study of Romanski (2007), the stimuli were vocalizations and faces, clearly more meaningful on the cognitive point of view than the simple noise bursts and LED stimuli delivered in the present behavioral paradigm.

In conclusion, our quantitative behavioral data, based on well controlled stimulation conditions, demonstrated in adult macaque monkeys that the detection of acoustic and visual stimuli presented simultaneously is faster and more reliable than when either stimulus is presented alone, in line with previously reported human data. In the PM, neuronal activity recorded during this detection task exhibited in parallel a statistically significant difference in the distribution of response patterns to the stimuli across the three modalities (visual alone, acoustic alone, and bimodal), in the sense of a decrease of inhibitory responses in the bimodal condition, as compared to unimodal condition. However, at that step, there is not yet demonstration of a causality relationship between the change of response patterns’ distribution and the behavioral effect observed in the bimodal condition, as compared to unimodal stimulation. The present study confirmed that non-human primates are high standard model in multisensory research, especially because of the possibilities of direct transfer of knowledge to humans. This was supported at different levels by similar results (behavioral, for example) compared to those obtained in humans or in other primate species. The present preliminary steps toward the ambitious goal of gaining access to the knowledge of multisensory integration should pave the way to revisit some neurological diseases [Alzheimer disease, specific language impairment (SLI) or attention syndrome deficit (ASD)] which have been shown to interfere with usual illusions (e.g., McGurk effect) when mismatching auditory and visual cues are presented. The outcome of such studies might elucidate the underlying mechanisms of unified percepts and therefore opening up new paths in clinical research regarding some still pending medical challenges.

## Conflict of Interest Statement

The authors declare that the research was conducted in the absence of any commercial or financial relationships that could be construed as a potential conflict of interest.

## Supplementary Material

The Supplementary Material for this article can be found online at http://www.frontiersin.org/Journal/10.3389/fnhum.2013.00799/abstract

## Abbreviations

RT, reaction time; PM, premotor cortex; PSTH, peristimulus time histogram; SD, standard deviation.
